# LysoPE mediated by respiratory microorganism *Aeromicrobium camelliae* alleviates H9N2 challenge in mice

**DOI:** 10.1186/s13567-024-01391-x

**Published:** 2024-10-11

**Authors:** Qingsong Yan, Junhong Xing, Ruonan Zou, Mingjie Sun, Boshi Zou, Yingjie Wang, Tianming Niu, Tong Yu, Haibin Huang, Wentao Yang, Chunwei Shi, Guilian Yang, Chunfeng Wang

**Affiliations:** College of Veterinary Medicine, Jilin Provincial Engineering Research Center of Animal Probiotics, Jilin Provincial Key Laboratory of Animal Microecology and Healthy Breeding, Engineering Research Center of Microecological Vaccines (Drugs) for Major Animal Diseases, Ministry of Education, Jilin Agricultural University, Changchun, 130118 China

**Keywords:** Influenza, respiratory microbiota, *Aeromicrobium camelliae*, LysoPE (16:0)

## Abstract

Influenza remains a severe respiratory illness that poses significant global health threats. Recent studies have identified distinct microbial communities within the respiratory tract, from nostrils to alveoli. This research explores specific anti-influenza respiratory microbes using a mouse model supported by 16S rDNA sequencing and untargeted metabolomics. The study found that transferring respiratory microbes from mice that survived H9N2 influenza to antibiotic-treated mice enhanced infection resistance. Notably, the levels of *Aeromicrobium* were significantly higher in the surviving mice. Mice pre-treated with antibiotics and then inoculated with *Aeromicrobium camelliae* showed reduced infection severity, as evidenced by decreased weight loss, higher survival rates, and lower lung viral titres. Metabolomic analysis revealed elevated LysoPE (16:0) levels in mildly infected mice. In vivo and in vitro experiments indicated that LysoPE (16:0) suppresses inducible nitric oxide synthase (INOS) and cyclooxygenase-2 (COX2) expression, enhancing anti-influenza defences. Our findings suggest that *Aeromicrobium camelliae* could serve as a potential agent for influenza prevention and a prognostic marker for influenza outcomes.

## Introduction

Influenza is an acute viral respiratory disease that poses a significant threat to human and animal health, with seasonal outbreaks impacting thousands of both each year. Influenza viruses are classified according to the antigenic properties of their surface glycoproteins, with four main types identified so far [1]. Among these, the influenza A virus is recognised as an avian influenza strain [2]. As members of the *Orthomyxoviridae* family, these viruses have a segmented negative-sense RNA genome that encodes ten core proteins and a varying number of accessory proteins [3, 4]. They are further divided into 18 HA and 11 NA subtypes based on their surface glycoproteins: HA and NA [5]. Certain subtypes can cause serious infectious diseases in animals, resulting in significant economic losses for the poultry industry. Notably, China has experienced a clear increase in avian influenza cases in recent years [6].

Moreover, the H9N2 subtype, first isolated from turkeys in Wisconsin, USA, in 1966, has since spread globally [7–10]. The virus is common in various avian species worldwide, mainly chickens and ducks, but it can also infect humans and other mammals, making it a zoonotic disease [11]. Certain strains of H9N2 have evolved to bind receptors necessary for human infection, leading to mild respiratory illnesses and posing a significant health risk [12]. Avian Influenza Viruses (AIV) are divided into two categories based on their pathogenicity in chickens and molecular markers in HA proteins: highly pathogenic avian influenza viruses (HPAIV) and low pathogenic avian influenza viruses (LPAIV), known for their high and low pathogenicity, respectively [13]. Despite the H9N2 strain being classified as an LPAIV, it can still interact with other pathogens, thus reducing poultry performance, causing severe clinical symptoms, and increasing mortality rates [14–17]. Moreover, H9N2 AIV has been isolated from various mammals and can transfer genetic material to other influenza subtypes such as H5, H1, or H7. This ability to transfer can potentially create new viruses with increased pathogenicity [18–20]. Therefore, the widespread presence of the H9N2 virus across species could lead to a new influenza pandemic, threatening human and animal health [21, 22].

Scientific advancements have led to the identification of distinct microbial communities within the gastrointestinal tract that play a crucial role in maintaining host health [23, 24]. Known as ‘next-generation probiotics’, these beneficial commensal microorganisms are increasingly used to restore a healthy balance within the gastrointestinal environment [25]. Bacteria such as *Clostridium butyricum*, *Bacillus salivarius*, and *Akkermansia municiphila* have been shown to regulate immune responses in this tract [26–28]. The respiratory system also contains a diverse microbiota, with variations in types and quantities across different sections [29]. Numerous studies have demonstrated the microbiota’s role in enhancing host immunity, improving nutrient absorption, and strengthening the intestinal barrier [30, 31]. Specific microbial communities have recently been found throughout the respiratory tract, from the nostrils to the alveoli [32]. Changes in the composition or functionality of the respiratory microbiota can decrease the population of beneficial bacteria, compromising the respiratory system’s ability to defend against pathogens [33]. Although research on respiratory microbiota is not as extensive as it is for gastrointestinal microbiota, some studies have highlighted the beneficial impact specific bacteria can have on respiratory health, which has opened avenues for exploring and identifying novel respiratory ‘next-generation probiotics’ [34].

Microorganisms play a crucial role in maintaining host health, but they can also promote disease through microbiota dysbiosis. In addition to their digestive functions, these microorganisms produce various vitamins and benefit the host by producing short-chain fatty acids (SCFAs) such as butyrate, propionate, and acetate [35]. Metabolites are classified into three main types: (1) those produced by the intestinal microbiota from dietary components, such as vitamin K; (2) those secreted by host cells and modified by the gut microbiota, like secondary bile acids; and (3) those formed through the intestinal microbiota’s anaerobic fermentation, exemplified by SCFAs [36–38]. The microbiota and its metabolites are critical in regulating host physiology and pathophysiology, influencing a wide range of metabolic, inflammatory, and even behavioural processes [39, 40]. Studies have shown that microbial metabolites can modulate the host’s immune responses [41, 42].

LysoPE is a lysophospholipid produced by phosphatidylethanolamine (PE) deacylation during hydrolysis by phospholipase A1/A2 [43]. It has been found to affect lipid accumulation and metabolism in human liver-derived cell lines [44]. LysoPE also acts as a neurotrophic activator through the mitogen-activated protein kinase signalling pathway in pheochromocytoma cells [45]. Recent research has demonstrated that LysoPE not only enhances neurite outgrowth but also protects cultured cortical neurons against glutamate toxicity. Furthermore, it inhibits lipopolysaccharide-induced polarisation of M1 macrophages in murine peritoneal macrophages [46–48].

Thus, investigating the protective effects of respiratory flora and their metabolites on host organisms is essential for developing novel anti-influenza agents.

## Materials and methods

### Bacteria, viruses, and animals

*Aeromicrobium camelliae* was obtained from the China Microbial Strain Collection and Management Centre, catalogued under strain number JCM 30952, and cultured aerobically at 37 °C for 24 h in a Lysogeny Broth (LB) liquid medium.

J774A.1 and MDCK cells are maintained in our laboratory.

Our laboratory also preserves the H9N2 strain [49]. The virus’s titre was determined using the half-maximal infective dose (H9N210^6.5^EID_50_) in chicken embryos. Specific-pathogen-free (SPF) six-week-old C57BL/6 female mice were sourced from Changchun Yis Experimental Animal Technology Co. The mice were housed with unlimited access to water and food at the SPF Animal Farming Center. All animal-related procedures were performed in strict compliance with the guidelines of the Animal Management and Ethics Committee of Jilin Agricultural University (JLAU20200704001).

### Statistical analysis

Data were analysed using GraphPad Prism 8.0 software. Pairwise comparisons were conducted with the Student’s *t*-test, and values are presented as mean ± SD. *p < 0.05, **p < 0.01, ***p < 0.001.

### Experimental design and sample collection

The experimental framework was divided into four groups: Experiment A for establishing the H9N2 mouse model, Experiment B for inoculating mice with respiratory microorganisms, Experiment C for microbial terpene administration, and Experiment D for LysoPE (16:0) administration.

Experiment A: Six-week-old female C57BL/6 mice were divided into two groups: the infected group (*n* = 20) and the control group (*n* = 5; NC). On day 0, mice were intranasally inoculated with H9N2 (20 µL/animal). Post-inoculation, we conducted daily monitoring for 15 days (from day 0 to day 14) to assess the weight and survival of the mice. Concurrently, lung tissue, blood, and bronchoalveolar lavage fluid were collected from the subjects on day five post-inoculation. Part of the lavage fluid was frozen at -80 ℃ for future 16S sequencing and non-targeted metabolomics analysis. The remainder of the lavage fluid was centrifuged at 6000 × *g* for 15 min, suspended in 10% glycerol, and stored at -80 ℃. Lung specimens were fixed in 4% formaldehyde, embedded in paraffin, and subjected to haematoxylin and eosin (H&E) staining for histopathological evaluation and immunohistochemistry. Based on post-inoculation, lung viral loads and body weight trajectories, mice were classified into severe (S) and mild (M) subsets.

Experiment B: The collected bronchoalveolar lavage fluid was centrifuged. Glycerin was replaced by phosphate-buffered saline (PBS) and stained with methylene blue to quantify live microorganisms. Resuspension in PBS at 1 × 10^6^ CFU/40 μL was prepared for subsequent use [50]. Twenty antibiotic (ATB) pre-treated mice were randomly divided into four groups (*n* = 5/group). These groups were Group M, the nasal toxicity group with irrigation solution (Donor M + H9N2), Group S, the nasal toxicity group with irrigation solution (Donor S + H9N2), the PBS-inoculated group (PBS + H9N2), and the control group (NC). Mice in the Donor M and Donor S groups received a one-day nasal drip, while those in the PBS + H9N2 group received an equivalent volume of PBS. One day after intranasal inoculation with H9N2 (20 µL/vessel), mice were monitored daily for body weight and survival for 15 days (post-infection day 0 to day 14). A concurrent experiment replicated sample collection as outlined in Experiment A.

Experiment C: Before the virulence test, *Aeromicrobium* was aerobically incubated for 24 h, centrifuged at 4000 × *g* for 4 min at 4 °C, washed three times with sterile PBS (pH = 7.4), and resuspended in PBS at 1 × 10^8^ CFU/40 μL for subsequent application. Fifteen ATB pre-treated mice were randomly assigned to three groups (*n* = 5/group). These were the control (NC), *Aeromicrobium*-inoculated group (Ae + H9N2), and PBS-inoculated group (PBS + H9N2). Mice in the Ae + H9N2 group received *Aeromicrobium camelliae* via nasal administration for five days, while those in the PBS + H9N2 group received an equivalent volume of PBS. Following this, both groups, excluding the control, were intranasally inoculated with H9N2 (20 µL per mouse), with subsequent monitoring of survival and body weights for 14 days post-infection. A parallel study was conducted to replicate sample collection as per Experiment A.

Experiment D: To assess the anti-influenza efficacy of LysoPE (LysoPE (16:0), purchased from Sigma-Aldrich), 15 ATB pre-treated mice were randomly divided into three groups (*n* = 5/group). These included the control group (NC), metabolite repletion attack group (LysoPE + H9N2), and Vehicle attack group (Vehicle + H9N2). Mice in the LysoPE + H9N2 group were given LysoPE (1 mg/kg; 20 µL) (1% DMSO dissolved in PBS) via nasal drip for five days. Mice in the Vehicle group received the same volume of PBS via nasal drip. Five days later, the remaining groups, excluding the control, were intranasally challenged with H9N2 (20 µL per mouse), and their survival and body weights were tracked for 14 days post-infection. Concurrently, a replicated study was undertaken for sample collection, as detailed in Experiment A.

### J774A.1 cell inflammation model establishment

J774A.1 cells were cultured in Dulbecco’s Modified Eagle Medium (DMEM) (1% double antibody, 5% serum) and inoculated onto 12-well plates (2 × 10^5^ cells/well) for 24 h. Cells were treated with lipopolysaccharide (LPS) at concentrations of 100, 500, and 1000 ng/mL for 6 h. RNA was extracted from the cells, reverse transcribed, and quantified via qRT-PCR.

### LysoPE inhibition of J774A.1 cell inflammatory response model

J774A.1 cells were cultured in DMEM medium (1% double antibody, 5% serum) and inoculated onto 12-well plates (2 × 10^5^ cells/well) for 24 h. Cells were pre-treated with LysoPE at concentrations of 1 µM, 10 µM, and 50 µM for 24 h, followed by 6-h stimulation with 1 µg/mL LPS. RNA was subsequently extracted, reverse transcribed, and quantified using qRT-PCR, with further analysis through protein blotting.

### Collection of mouse alveolar macrophages

Alveolar macrophages (AMs) were isolated via BALF (bronchoalveolar lavage fluid). Following the euthanasia of the mice, the lungs were lavaged with Hanks’ balanced salt solution (HBSS). This process involved the slow injection and withdrawal of 2 mL of warm (37 °C) Ca2 + /Mg2 + -free HBSS (pH 7.4) with EDTA (0.6 mM), which was performed eight times [51]. The lavage fluid was collected and centrifuged (400 × *g*, 10 min, 4 °C), and the cells were prepared for subsequent applications.

### Antibiotic-treated mice

Following the procedures used in previous studies, the mice received one week of intranasal administration of 50 µL ddH2O containing ampicillin (1 mg/mL), vancomycin (0.5 mg/mL), metronidazole (1 mg/mL), and neomycin (1 mg/mL) to eradicate respiratory microbiota [52].

### 16srDNA sequencing

The hexadecyltrimethylammonium bromide (CTAB) method was used to extract the total microbiome DNA from various sample origins. We assessed the DNA quality by running it through agarose gel electrophoresis and using a UV spectrophotometer for quantification. To delineate the taxonomic composition of the mouse respiratory microbiome, the V3-V4 region of the 16 S rDNA gene was targeted for sequencing with commonly used primers (F: 5′-CCTACGGGNGGCWGCAG-3′; R: 5′-GACTACHVGGGTATCTAATCC-3′). Amplification reactions were prepared using 50 ng of template DNA in a total volume of 25 μL. PCR conditions included an initial denaturation at 98 °C for 30 s, followed by 35 cycles of 10 s at 98 °C, 30 s at 54 °C, and 45 s at 72 °C, with a final extension of 10 min at 72 °C.

Following purification, we evaluated the PCR products using the Agilent 2100 Bioanalyzer (Agilent, USA) in conjunction with Illumina library quantification kits (Kapa Biosciences, Woburn, MA, USA). Acceptable library concentrations were established at a minimum of 2 nM. Qualifying libraries, ensuring non-repetitive index sequences, were diluted based on the required sequencing depth, denatured to single strands with NaOH, and prepared for sequencing. A 2 × 250 bp paired-end sequencing protocol was executed on the NovaSeq 6000 platform.

Diversity analysis was divided into alpha and beta diversity assessments based on the procured Amplicon Sequence Variant (ASV) sequences and corresponding abundance tables. Alpha diversity within the microbial communities was quantified using six indices: Observed_species, Shannon, Simpson, Chao1, Goods_coverage, and Pielou_e. Beta diversity was assessed by calculating four distances to assess diversity between habitats (between samples/groups), using six main analyses (Weighted_unifrac, Unweighted_unifrac, Jaccard, Bray_curtis).

Species Annotation: Using the SILVA database, species were identified based on the ASV sequences and further annotated with the NT-16S database. Subsequently, the abundance of each species within individual samples was enumerated based on the ASV abundance table.

Variance and Subsequent Analysis: The discrepancies between comparative groups were analysed based on the species abundance data retrieved. The selection of an appropriate statistical method was contingent upon the sample specifics. In this study, the Kruskal–Wallis test was employed to distinguish the differences among multiple groups, each comprising biological replicates of the samples (*p* < 0.05).

### Non-targeted metabolomics analysis

The acquired liquid samples were initially thawed on ice, and 20 µL of each sample was extracted with 120 µL of pre-cooled 50% methanol. The samples were vortexed for 1 min, incubated at room temperature for 10 min, and stored overnight at −20 °C. Following centrifugation at 4000 *g* for 20 min, the supernatant was transferred to a fresh 96-well plate and preserved at −80 °C prior to LC–MS analysis. Concurrently, quality control (QC) samples were prepared by combining 10 µL from each extract. After sample preparation, the extracts underwent systematic analysis. Initially, the raw mass spectral data were converted into an interpretable format using Proteowizard msConvert software, converting to mzXML. XCMS software facilitated peak extraction, which was then subjected to QC. CAMERA was used for compound annotation and preliminary identification via metaX software. This identification included Level 1 mass spectrometry information, and Level 2 mass spectrometry data correlated with an in-house standards database. The putative identifiers were further annotated using databases such as HMDB and KEGG, which elucidate the metabolites’ physicochemical properties and biological functions. Differential metabolites were quantified and screened using metaX software.

### Analysis of cytokine concentrations

The blood samples were allowed to clot overnight at 4 °C, followed by centrifugation to obtain the serum (1000 rpm, 4 °C, 5 min). The lung tissues were homogenised in PBS at a 1 mL/0.1 g lung ratio, and the homogenate was clarified by centrifugation (3000 rpm, 4 °C, 5 min). Cytokine levels, specifically TNF-α, IL-1β, and IL-6, were assessed using ELISA kits according to the manufacturer’s protocol. The absorbance was calculated at 450 nm with an enzyme-linked immunosorbent assay reader, and cytokine concentrations were deduced from a standard curve.

### Virus titre detection

To determine the virus titre and obtain a serially diluted tenfold suspension of tissue homogenate, an equal weight of lung tissue was homogenised in DMEM. It was then titrated in 96-well culture plates of MDCK cells. The Reed-Muench method was adopted to calculate the titre, which was expressed as log_10_TCID_50_/g of lung tissue [53].

### Histological analysis

The mouse lung tissues were fixed in 4% formaldehyde, embedded in paraffin, and sectioned. Each sample comprised at least two tissue section (3 μm). These sections were heated in an oven at 80 °C for 1 h, then cooled to room temperature. After the haematoxylin–eosin (H&E) staining, the sections were observed through an inverted fluorescence microscope (Leica Microsystems, Germany).

### Immunohistochemical analysis

The lung tissue was fixed in 4% formaldehyde, paraffin-embedded, and sectioned. Each sample contained at least two tissue sections of 3 μm thickness. Sections were then baked at 80 °C for 2 h, dewaxed and immersed in antigen retrieval solution (1 ×; sodium citrate antigen repair solution 50 ×, diluted with double distilled water). The solution was preheated to 95 °C-100 °C, and sections were heated for approximately 20 min at this temperature. After cooling, sections were rinsed twice with PBS for 5 min each. The tissue perimeters were circumscribed using a 0.3% Triton-100 pen, allowing for membrane permeabilisation. They were then incubated in darkness at room temperature for 60 min.

After discarding the liquid and blotting, sections were incubated with the diluted primary antibody (H9N2 monoclonal sheep anti-rabbit antibody purchased from SinoBiological; 1:1000 PBS dilution) overnight at 4 °C in the light. On the following day, the sections were washed three times with PBS for 5 min each. After blotting, sections were incubated with the secondary antibody (H9N2 secondary antibody sheep anti-rabbit purchased from Servicebio; 1:600 PBS dilution) and incubated for 1 h at room temperature. They were then washed twice with PBS for 5 min each. DAPI (1:1000 PBS dilution) was applied for 5 min, protected from light, followed by three PBS washes for another 5 min each. Sections were finally analysed using an inverted fluorescence microscope (Leica Microsystems, Germany).

### Quantitative real-time PCR

Using the TaKaRa MiniBEST Universal RNA Extraction Kit, total RNA was extracted from treated J774A.1 cells and mouse AMs. Complementary DNA (cDNA) was synthesised from approximately 2 µg of total RNA using the SweScript All-in-One RT SuperMix for qPCR Synthesis Kit (Servicebio). The mRNA expression of TNF-α, IL-1β, IL-6, INOS and COX was analysed on a real-time fluorescent quantitative PCR assay system using the SYBR green premix kit (Servicebio). mRNA expression levels were calculated using the 2^−ΔΔCt^ method and normalised to Glyceraldehyde-3-Phosphate Dehydrogenase (GAPDH). The primer sequences are as follows: mouse TNF-α, forward 5′-TTGTCTACTCCCAGGTTCTCT-3′ reverse 5′- GAGGTTGACTTTCTCCTGGTATG-3′; mouse IL-6, forward 5′-CTGCAAGAG ACTTCCATCCAG-3′, reverse 5′-AGTGGTATAGACAGGTCTGTTGG; mouse IL-1β forward 5′-AACCTGCTGGTGTGTGACGTT C-3′, reverse 5′-CAGCACGAG GCTTTTTTGTTGT-3′; mouse INOS forward 5′-GGAATCTTGGAGCGAGTTGT-3′, reverse 5′-CCTCTTGTCTTTGACCCAGTAG-3′, mouse COX-2, forward 5′- CGGACTGGATTCTATGGTGAAA-3′, reverse 5′-CTTGAAGTGGGTCAGGATGTAG-3′; mouse GAPDH, forward 5′-AACGTGTCAGTCGTGGACCTG-3′, reverse 5′-AGT GGGTGTCGCTGTFGAAGT-3′.

### Protein blotting analysis

The cells were centrifuged in RIPA buffer (Beyotime) containing PMSF (Servicebio), totipotent nuclease (Servicebio), and phosphatase inhibitor (Servicebio) at 3500 rpm for 2 min to collect the cell supernatant. We used the BCA protein assay kit (Beyotime) to determine protein concentrations. The protein samples (10 μg/lane) underwent separation via MOPS-PAGE and were then transferred to a polyvinylidene difluoride (PVDF) membrane. The membranes were blocked using rapid blocking solution (Beyotime) for 30 min at room temperature before overnight incubation with antibodies targeting INOS, COX-2, and GAPDH (Proteintech) at 4 °C. Thereafter, the membrane was exposed to the relevant horseradish peroxidase-linked secondary antibody (Servicebio) for 1 h at room temperature. Protein bands were then made visible using an enhanced chemiluminescence (ECL) system.

## Results

### C57bl/6 mice differentially targeted for H9N2 infection

**Figure 1:**
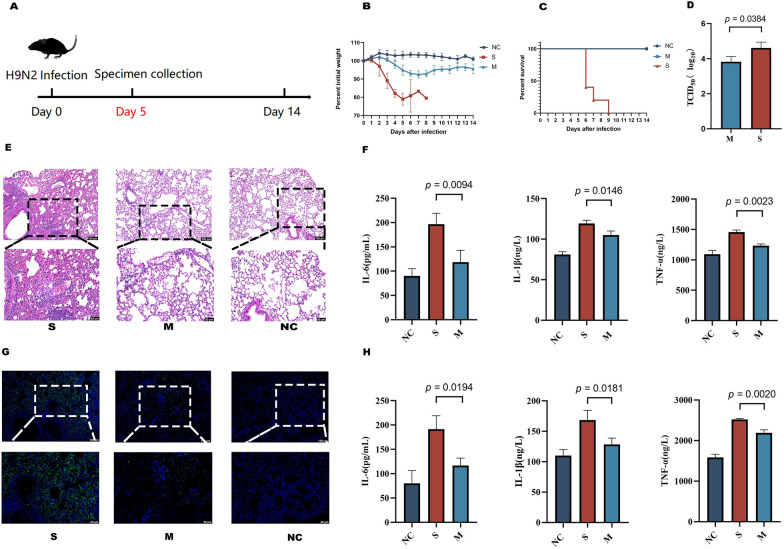
**Mice exhibit a heterogeneous response to influenza infection.** **A** Experimental procedure. **B** Variability in body weight. **C** Survival rates. **D** Pulmonary virus load (log_10_TCID_50/g_ of lung tissue). **E** H&E staining of mouse lung tissue. **F** Serum cytokine levels. **G** Immunohistochemical analysis of lung tissue. **H** Lung tissue cytokine levels. Data are presented as mean ± SD. S: Severe infection group; M: Mild infection group.

The above processes found that mice infected with H9N2 exhibited different clinical symptoms. For example, all mice in Group S displayed pronounced signs of infection, including weight loss, anorexia, and a coarse coat (Figure 1B and C). All mice from the NC and M groups survived, with the NC group showing no evident signs of infection. Mice in Group M exhibited fewer signs of infection than those in Group S. The virus titre test results indicated that the pulmonary virus titre in Group M was significantly lower than in Group S (Figure 1D). The histopathological and immunohistochemical evaluations revealed that the M group had less severe lung lesions and a lower viral load than Group S (Figure 1E and G). The ELISA assay results showed higher levels of inflammatory cytokines TNF-α, IL-1β, and IL-6 in the lungs and serum of Group S compared to the other two groups (Figure 1F and H).

### Transfer of respiratory microbiota in mice

**Figure 2:**
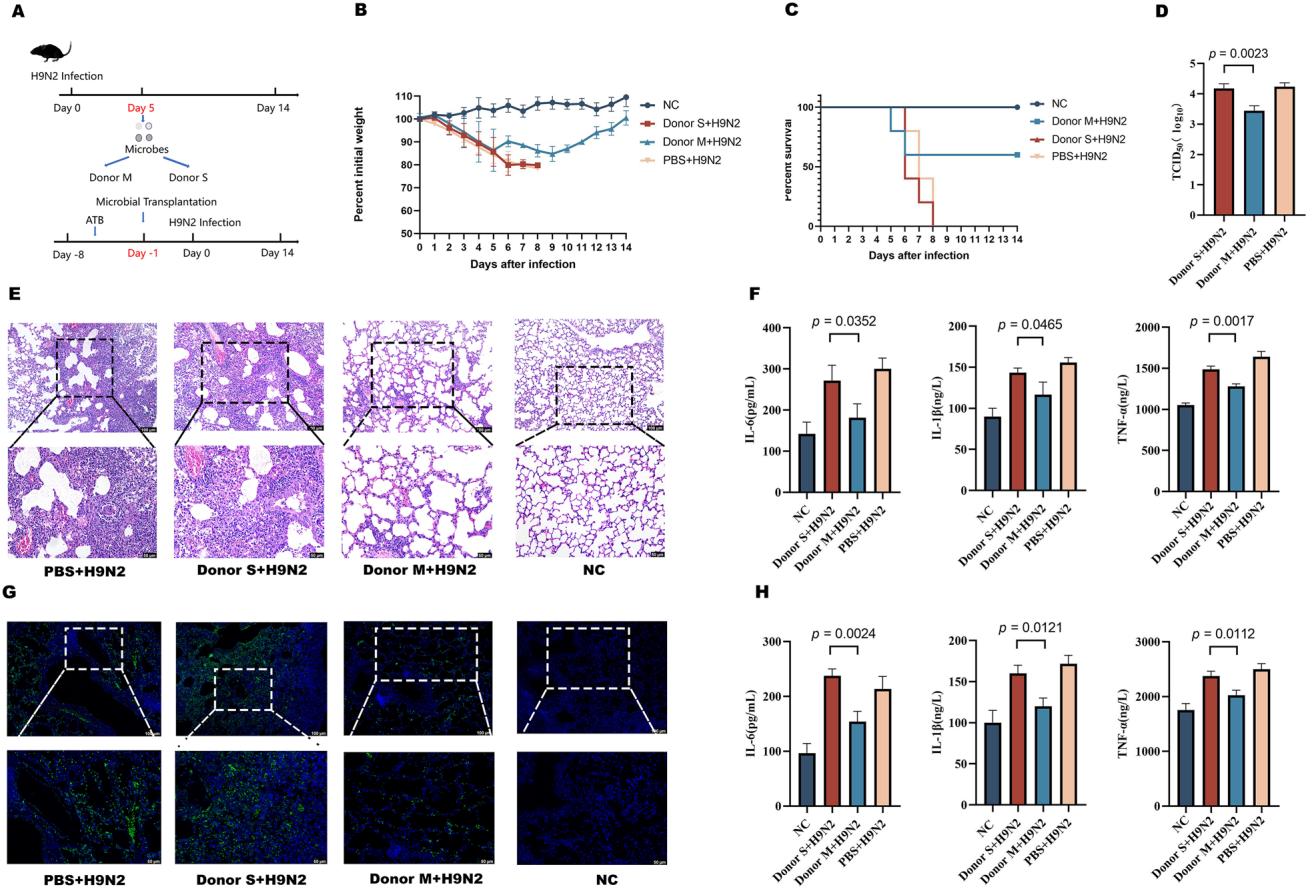
**Variability in response to influenza due to differences in respiratory microbiota in mice. A** Experimental procedure. **B** Changes in body weight. **C** Survival rates. **D** Viral load in lungs (log_10_TCID_50/g_ of tissue). **E** H&E staining of lungs. **F** Levels of cytokines in serum. **G** Immunohistochemical evaluations of the lungs. **H** Cytokine levels in lung tissues. Data are expressed as mean ± SD. Donor M: transfer from mild infection group; Donor S: transfer from severe infection group.

We conducted respiratory microbiota transplants to determine if differential mouse responses to influenza infection correlated with variations in respiratory microbiota. We then evaluated the impact of respiratory microbiota transferred from mice in groups S and M on viral infection in recipient mice (Figure 2A). The data showed that transferring respiratory microbiota from Group M mice to recipients on the fifth day after infection augmented their chances of survival.

Conversely, recipients that received respiratory microbiota from Group S displayed heightened infection rates (Figure 2B and C). The results of virus titre detection showed that the lung virus titre in the Donor M group was significantly lower than in the Donor S group (Figure 2D). Histopathological and immunohistochemical analyses revealed fewer lung pathologies and a reduced lung viral load in the Donor M group than in the Donor S group (Figure 2E and G). ELISA results indicated that cytokines TNF-α, IL-1β, and IL-6 levels were more pronounced in both serum and lungs of mice from the Donor S group than in the remaining groups (Figure 2F and H). These findings suggest that the respiratory microbiota in mice that survived the infection may contain specific respiratory microbes that provide protection against influenza.

### The respiratory microbiota exhibits distinctive characteristics depending on the severity of the influenza infection

**Figure 3:**
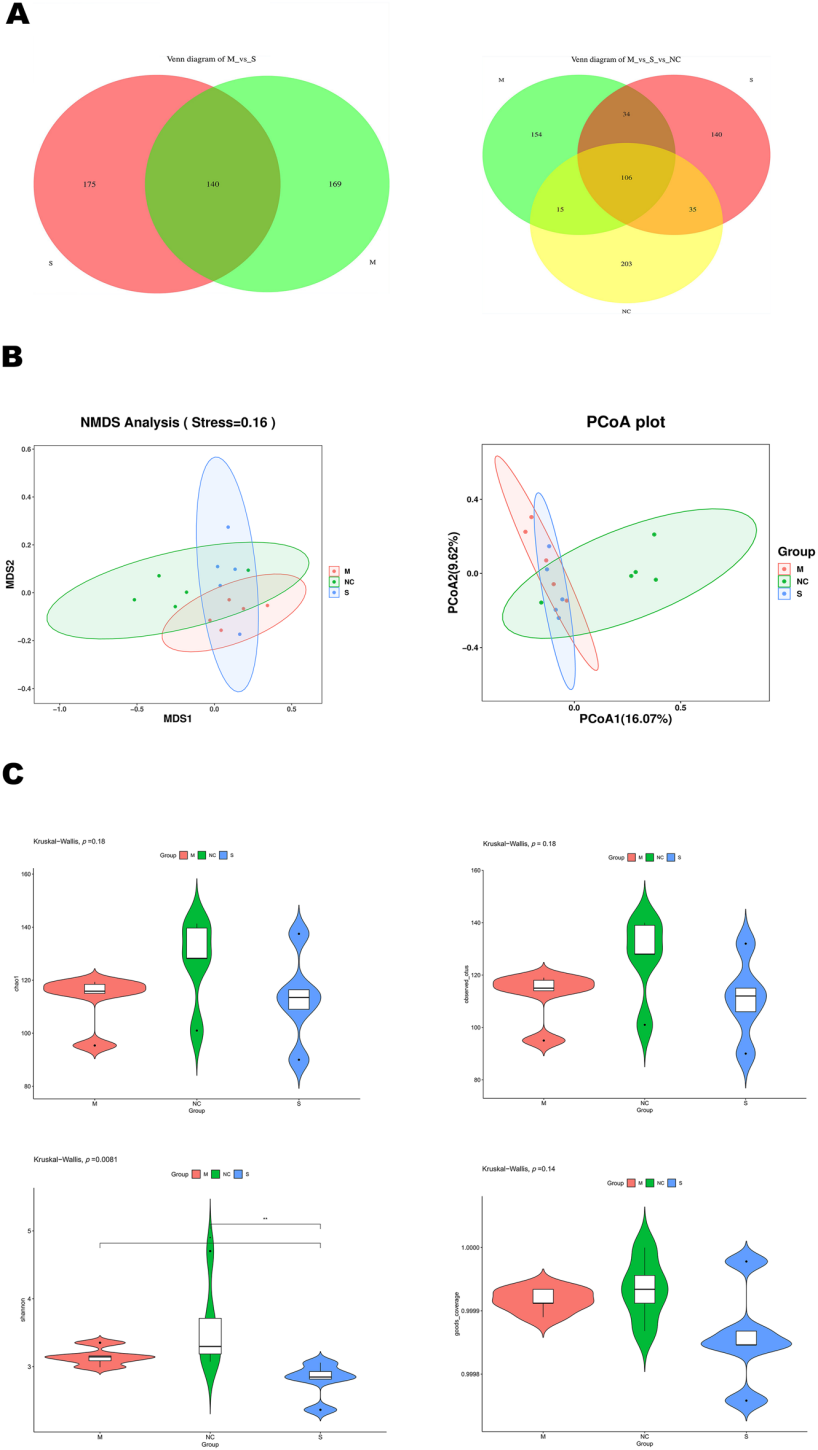
**Diversity of respiratory microbiota in mice analysed via alveolar lavage fluid. A** Venn diagram showing shared and unique ASVs (species) derived from abundance tables. **B** PCoA and NMDS plots based on the Jaccard distance matrix for groups M and S. **C** Violin plots of subgroup differences analysed by Kruskal–Wallis test.

To explore influenza-resistant respiratory microbes in mice that survived the infection, 16S rDNA sequencing was used to assess the respiratory microbiota of mice from the S, M, and NC groups on the fifth day of the study. The findings show that the species abundance table exposed marked variances in species count between the M and S groups (Figure 3A). Furthermore, the alpha diversity analysis results indicated that the NC group had the highest species count and greater diversity than the M and S groups. However, the M group maintained greater species count and diversity than the S group (Figure 3C). Both principal coordinate analysis (PCoA) and non-metric multidimensional scaling (NMDS) analyses pinpointed disparities in species composition between the M and S groups (Figure 3B).

**Figure 4:**
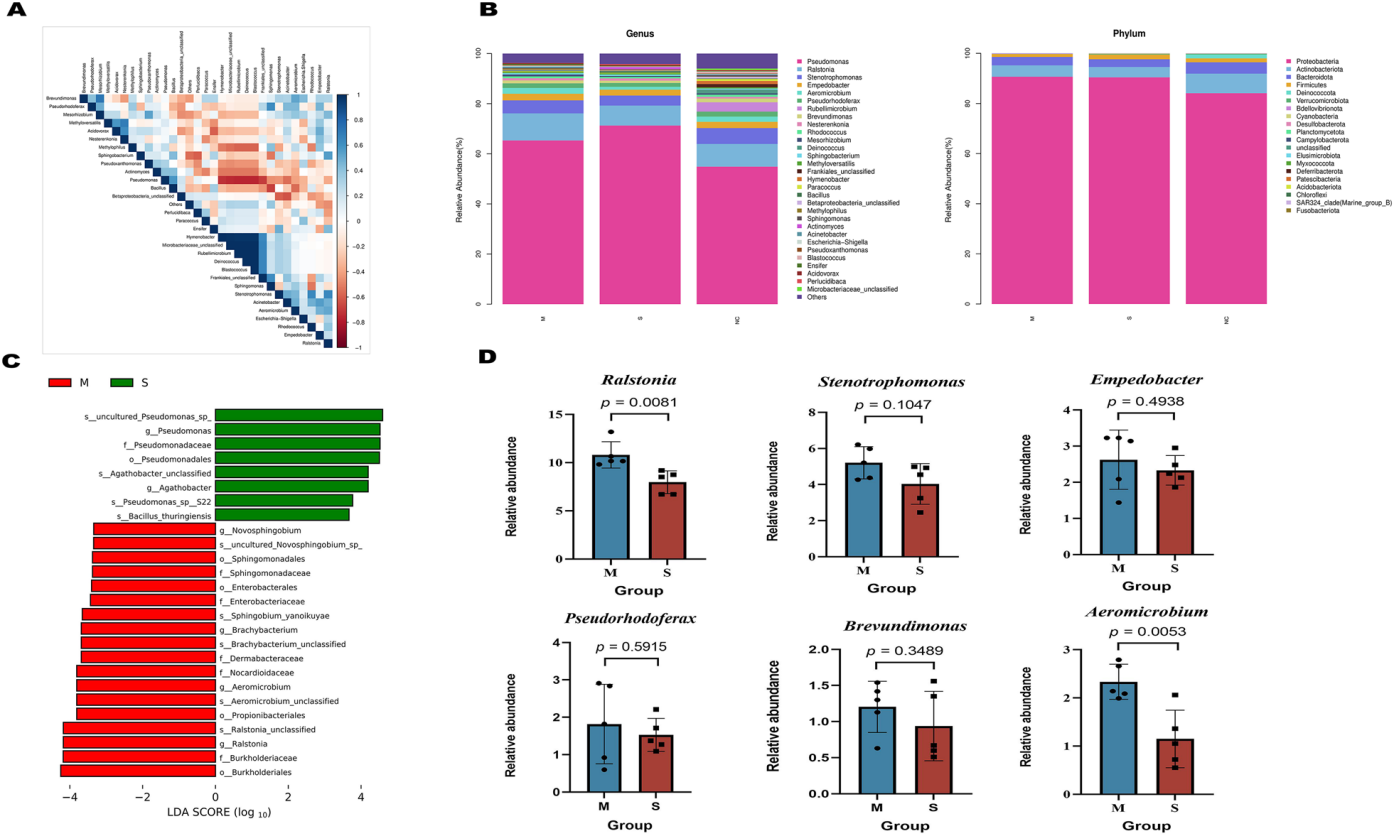
**Comparative analysis of microbial diversity in mouse respiratory systems.** **A** Correlation heatmap (via Spearman’s rank correlation) illustrating relationships among the top 30 microorganisms at the genus level, highlighting correlations and significance (*P*-value). **B** Relative abundance of bacterial phyla and genera across groups M, S, and NC. **C** LEfSe analysis showing differences in species between groups M and S five days post-infection. D. Comparison of the six most abundant genera using Student’s *t*-test.

Furthermore, when considering the phylum level, it was observed that the respiratory microorganisms’ composition in all three groups predominantly consisted of *Proteobacteria*, *Actinobacteria*, *Bacteroidota*, *Firmicutes*, and *Deinococcota*. Notably, the presence of *Proteobacteria* was significantly greater in the M and S groups than in the NC group. From the point of view of genera, *Pseudomonas* spp., *Ralstonia* spp., *Stenotrophomonas* spp., *Empedobacter* spp., and *Aeromicrobium* spp. dominated, and the abundance of *Pseudomonas* spp., was significantly higher in group S than that of group NC and group M. *Ralstonia* spp., *Stenotrophomonas* spp., *Empedobacter* spp., and *Aeromicrobium* spp. were more abundant in group M than that of group S (Figure 4B). The LEfSe variance analysis revealed that certain species, such as *Pseudomonas*, *Bacillus thuringiensis*, and *Agathobacter recti,* were particularly distinct in Group M. Meanwhile, in Group S, *Burkholderia*, *Ralstonia*, *Propionibacteriales*, and *Nocardioidaceae* were distinct (Figure 4C). The analysis of the six most prevalent genera revealed significant differences in the respiratory microbiota of mice that exhibited varying symptoms post-influenza infection (Figure 4D). We hypothesise that the genus *Aeromicrobium* may play a role in bolstering host defences against influenza.

### *Aeromicrobium camelliae* is strongly associated with the viability of influenza-infected mice

**Figure 5:**
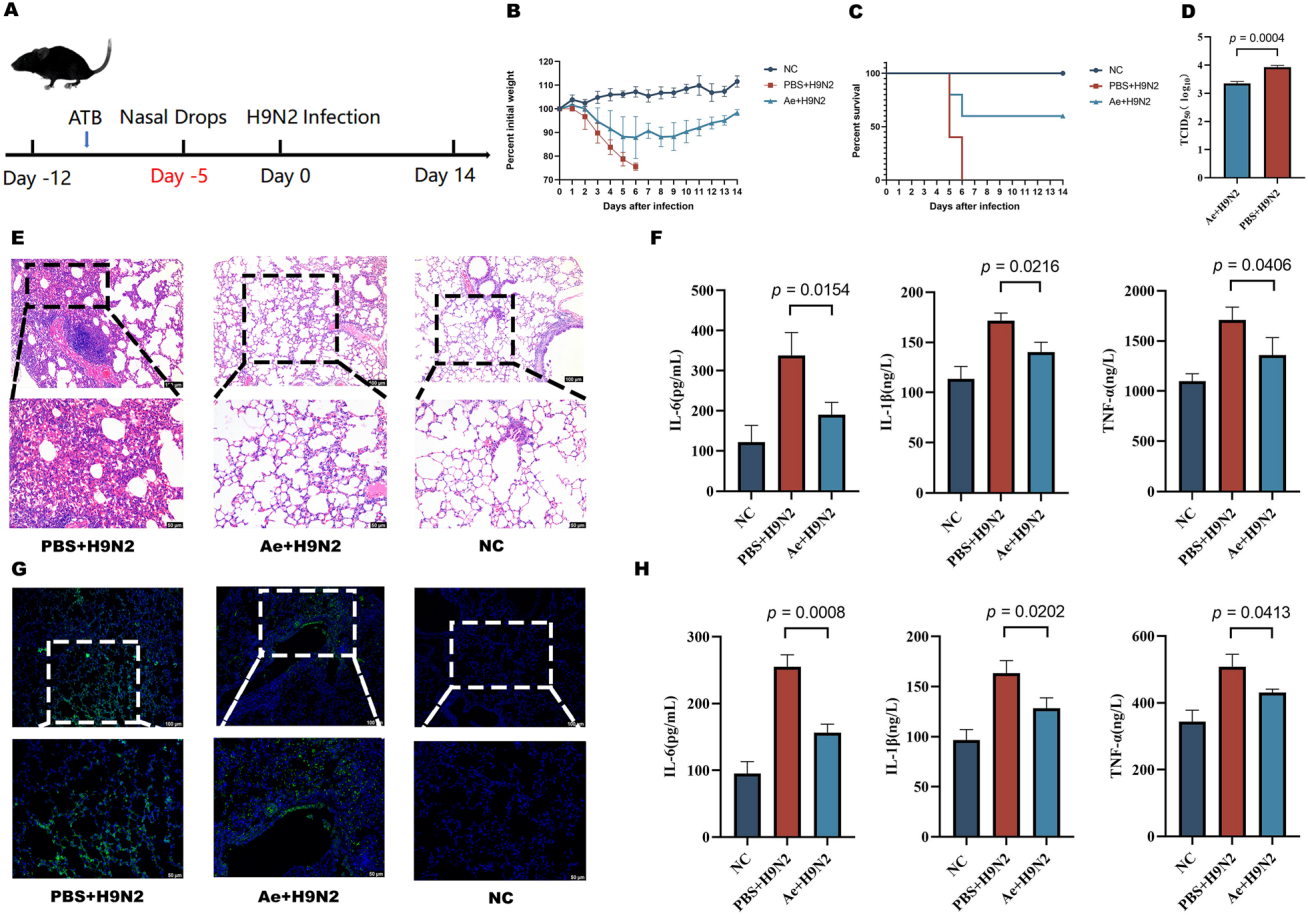
**The genus* Aeromicrobium* and its potential impact on diverse influenza responses in mice. A** Experimental procedure. **B** Body weight variability. **C** Survival rates. **D** Pulmonary virus concentration (log_10_TCID_50/g_ of lung tissue). **E** H&E staining of lung tissues. **F** Serum cytokine concentrations. **G** Immunohistochemical analysis of lungs. **H** Cytokine levels in lung tissues. Data are presented as mean ± SD. PBS + H9N2: Control group; Ae + H9N2: Bacterial challenge group.

In this study, mice were subjected to an ATB solution to investigate the influence of *Aeromicrobium camelliae* on their resistance to influenza. Subsequently, *Aeromicrobium camelliae* was administered nasally prior to the infection (Figure 5A). Data suggested that *Aeromicrobium camelliae* markedly increased the survival rates of the recipient mice (Figure 5B and C). Control experiments, utilising ATB-prepped mice, were conducted to ascertain cytokine levels and scrutinise histological alterations and immunohistochemistry. On the fifth day post-infection, the bulk of the cytokines in *Aeromicrobium camelliae*-administered mice presented a significant surge in both groups (Figure 5F and H). Notably, cytokines TNF-α, IL-1β, and IL-6 registered lower levels in the serum and lungs of mice treated with *Aeromicrobium camelliae* than those treated with PBS. This outcome infers that *Aeromicrobium camelliae* might fortify the host against influenza by fine-tuning its immune mechanism. The results of the virus titre test showed that the lung virus titre of the *Aeromicrobium camelliae* group was significantly lower than that of the PBS group (Figure 5D).

Furthermore, histological evaluations showed that on the fifth day after infection, the group that received PBS demonstrated significant pathological changes, including congestion, increased inflammatory cells, and a near-complete loss of alveolar structure. Conversely, those treated with *Aeromicrobium camelliae* demonstrated milder lung pathologies (Figure 5E). Immunohistochemical analyses further reinforced a decline in lung viral load for mice exhibiting a milder infection post *Aeromicrobium camelliae* administration (Figure 5G). This finding solidifies the proposition that *Aeromicrobium camelliae* potentially shields the host from influenza by modulating its immune response.

### *Aeromicrobium* may protect hosts against influenza viruses by regulating respiratory microbial metabolism

**Figure 6:**
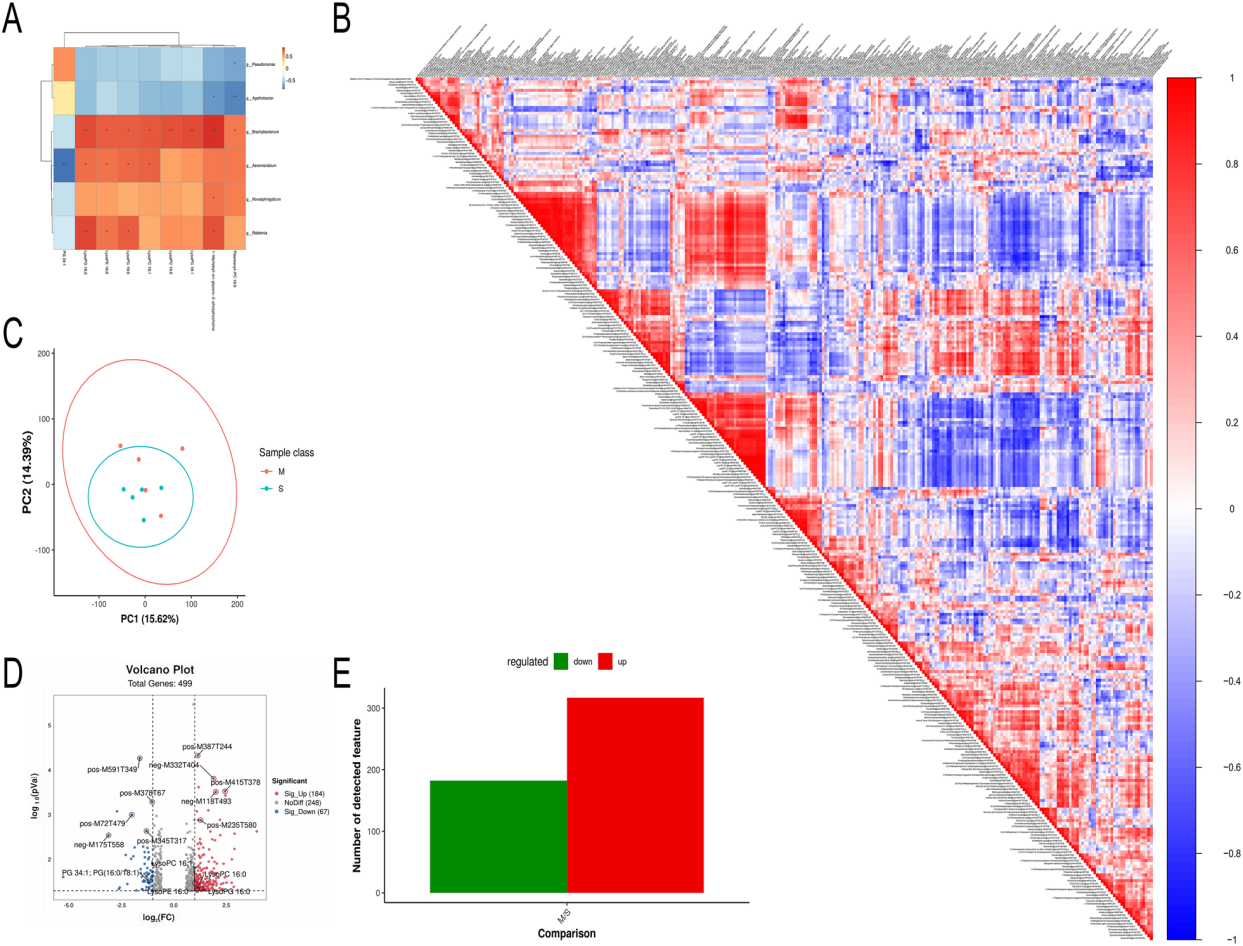
**Extensive metabolomic analysis of alveolar lavage fluid in mice.** **A** Correlation graphs show associations between different genera and metabolites (via Spearman’s correlation coefficient) integrating untargeted metabolomics with 16 s co-analysis. **B** Analysis of metabolites (post-normalisation) identified by ion profiles (red for positive correlations, blue for negative; the intensity of the colour indicates stronger correlation). **C** Principal Component Analysis (PCA) illustrates variability within and between groups. **D** Volcano plots, utilising differences in metabolic ions as the horizontal axis and -log_10_ (q-value) as the vertical axis, aid in identifying significant metabolic variations. **E** Univariate analysis using fold-change and the t-test, enhanced by BH Correction for q-value and Variable Importance in Projection (VIP) scores from PLS-DA, identifies and highlights differentially expressed metabolic ions, culminating in a differential ion histogram.

We conducted an analysis of respiratory microorganism metabolites using untargeted metabolomics techniques. The PCA plot reveals distinct variability in metabolic ions between the M and S groups (Figure 6C). The correlation heatmap indicates a significant positive correlation for LysoPE 16:0 (Figure 6B). Moreover, the differential ion histogram demonstrates the occurrence of both up-regulated and down-regulated differential ions in the M and S groups (Figure 6E). Metabolites such as LysoPE 16:0, LysoPG 16:0, LysoPC 16:1, LysoPC 16:0, and PG 34.1; PG (16:0/18:1) were identified (Figure 6D). These metabolites might contribute to the influenza resistance observed in the Donor M group of respiratory microorganisms. Upon integrating metabolomics and 16S in a multi-omics approach, differences between *Aeromicrobium* and LysoPE (16:0) emerged (Figure 6A). We hypothesise that *Aeromicrobium* may fortify hosts against influenza viruses through modulation of respiratory microbial metabolism.

### LysoPE protects hosts against influenza infection

**Figure 7:**
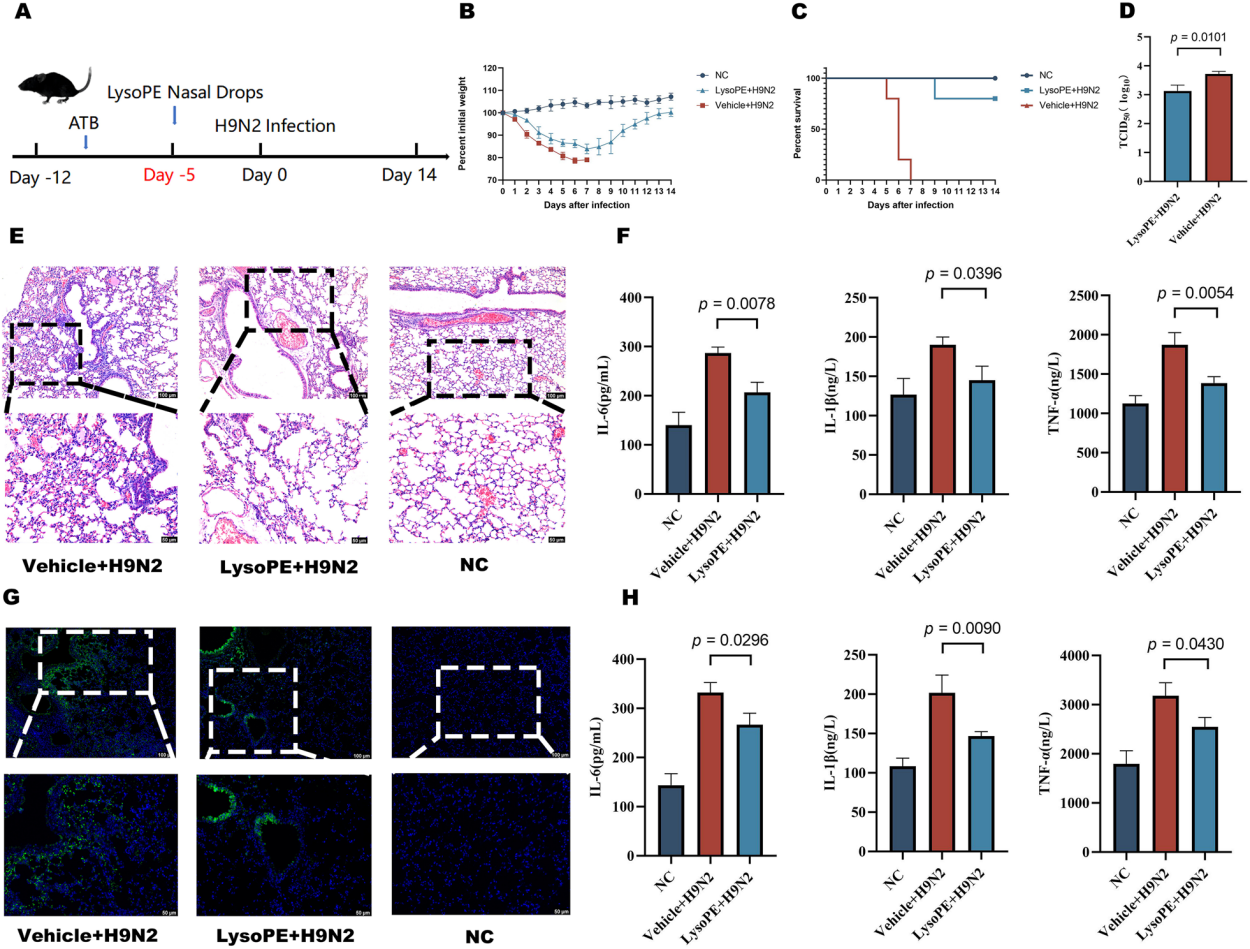
**Potential influence of the respiratory microbial metabolite LysoPE on heterogeneous influenza responses in mice.** **A** Experimental procedure. **B** Variations in body weight. **C** Survival rate of mice. **D**. Viral load in lung tissue (log_10_TCID_50/g_). **E** H&E staining of lung tissues. **F** Serum cytokine levels. **G** Immunohistochemical analysis of lung tissues. H. Cytokine levels in lung tissues. Data are presented as mean ± SD. Vehicle + H9N2: Control group; LysoPE + H9N2: Metabolite challenge group.

To ascertain whether LysoPE can defend against influenza infection in mice, LysoPE was administered, as previously mentioned, to ATB-treated mice through nasal drops. Subsequently, mice were inoculated intranasally with the H9N2 virus (Figure 7A). As depicted in the graph, mice that received LysoPE exhibited enhanced survival rates and mitigated weight loss (Figure 7B and C). According to the virus titre test results, the lung virus titre of the LysoPE group was significantly lower than that of the Vehicle group (Figure 7D). In vivo*,* complementary animal studies were executed to investigate LysoPE’s anti-influenza mechanism more deeply. On the fifth day after infection, we collected samples of lung tissue, serum, and alveolar lavage fluid to measure cytokine levels, evaluate histological changes, and perform immunohistochemical assays. Notably, the concentrations of inflammatory cytokines TNF-α, IL-1β, and IL-6 were diminished in the serum and lungs of the LysoPE-treated mice on day five post-infection (Figure 7F and H). Histological evaluations coupled with immunohistochemical analyses further revealed that LysoPE treatment curtailed tissue damage and lessened the viral burden in the lungs (Figure 7E and G).

**Figure 8:**
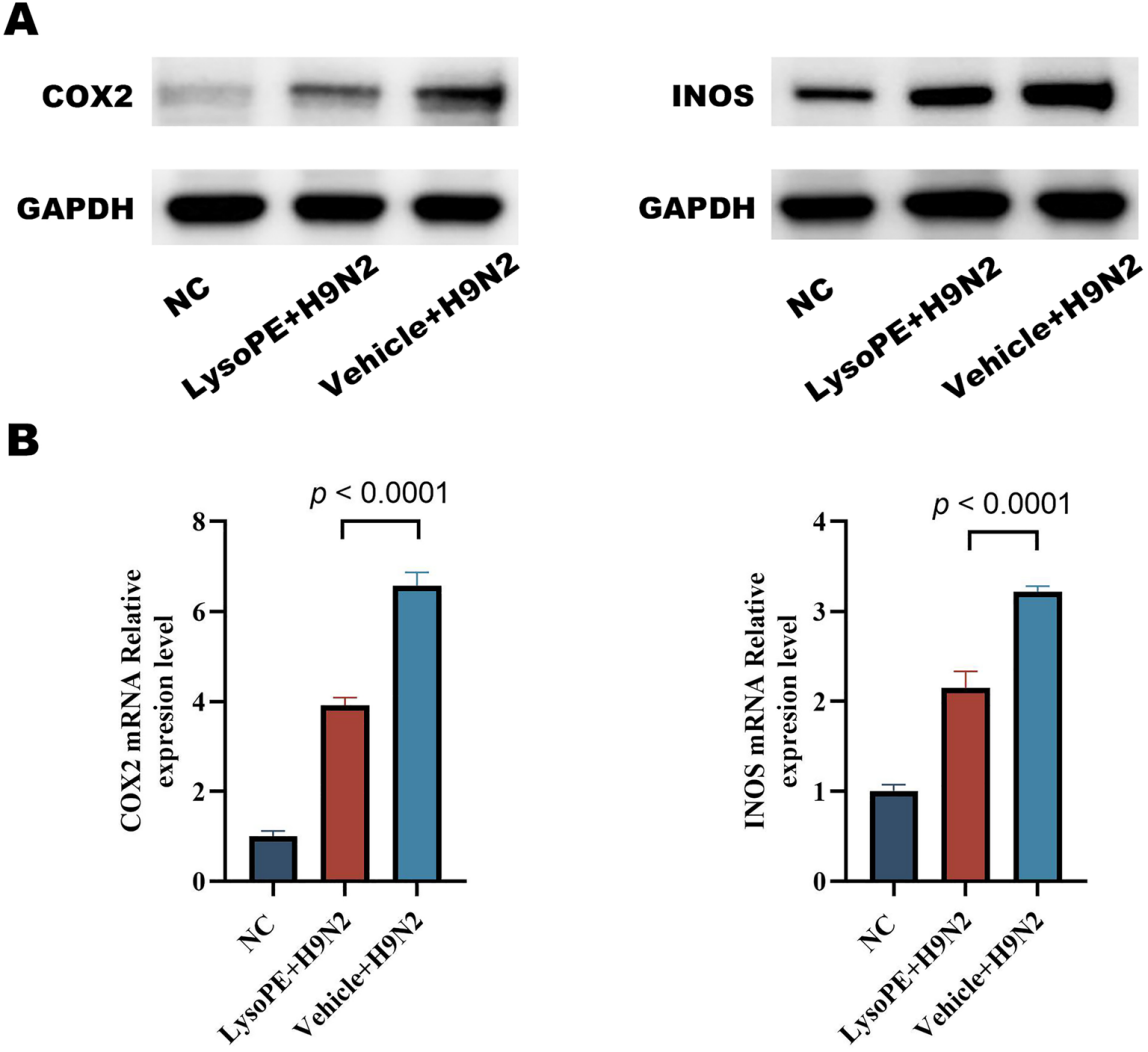
**Variability in influenza response in mice possibly due to respiratory microbial metabolite LysoPE.** **A** Protein expression levels of INOS and COX-2 in alveolar macrophages. **B** mRNA transcription levels of INOS and COX-2 in alveolar macrophages. Data are expressed as mean ± SD from three independent experiments.

Western blot analysis and qRT-PCR of isolated AMs showed decreased protein expression levels and transcription levels of INOS and COX-2 genes in LysoPE-treated mice compared with the Vehicle group (Figure 8A and B). As pivotal inflammatory mediators, INOS and COX-2 often serve as markers for gauging inflammation severity. This result underscores the potential of LysoPE to temper systemic inflammation, most likely through inhibiting INOS and COX-2 expression.

### Effect of LPS stimulation on transcript levels of cytokines associated with J774A.1 cells

**Figure 9:**
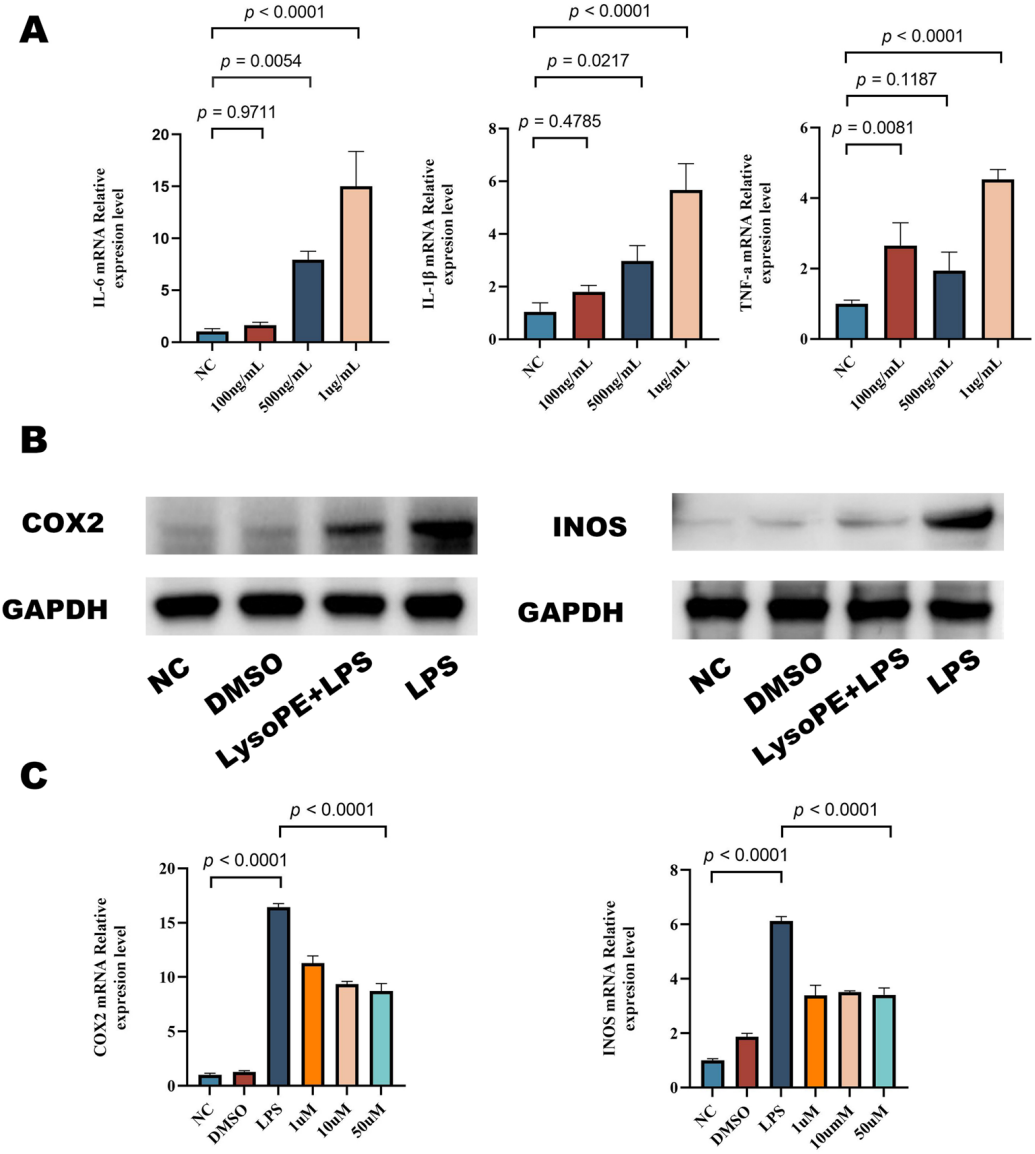
**LysoPE reduces LPS-induced expression of INOS and COX-2 in J774A.1 cells.** **A** Gene expression of IL-6, TNF-α, IL-1β in J774A.1 cells post-exposure to various LPS concentrations for 6 h. **B** Protein expression levels of INOS and COX-2 after 24 h exposure to LPS following 50 µM LysoPE pretreatment. **C** mRNA transcription levels of INOS and COX-2 in LPS-stimulated cells after 24 h pretreatment with 50 µM LysoPE. Data are presented as mean ± SD from three independent experiments.

J774A.1 cells were exposed to varying concentrations (100, 500, 1000 ng/mL) of LPS for 6 h. Real-time quantification results manifested that gene expression of inflammation-associated markers TNF-α, IL-1β, and IL-6 stimulated with 1ug/mL LPS was higher than that of the control group (Figure 9A).

### LysoPE inhibits LPS-induced expression of INOS and COX-2 in J774A.1 cells

We employed qRT-PCR and protein blotting to assess the influence of LysoPE on INOS and COX-2 expression in J774A.1 cells. The results indicated that LPS treatment amplified mRNA expression of INOS and COX-2 in J774A.1 cells. However, when pre-treated with LysoPE, LPS-induced INOS and COX-2 expressions were significantly reduced (Figure 9B and C).

## Discussion

We present evidence that *Aeromicrobium camelliae* offers protective effects against the H9N2 influenza virus in mice. Furthermore, intranasal administration of LysoPE (16:0), a metabolite of respiratory microbiota, diminished the severity of influenza virus infections in mice.

Recent scientific research has increasingly emphasised the benefits of bacterial colonisation on mucosal surfaces across a range of fields, including nutrition, immunology, and behavioural studies. Historically, the lungs were presumed to be sterile environments. This presumption directed most research attention to the densely colonised gut microbiota. As such, limited data exist on the lung microbiota’s role in regulating immunity and maintaining homeostasis in vivo [54–57]. Scientific methodologies have advanced significantly, particularly with the high-throughput sequencing of the 16S gene, which has greatly expanded our understanding of this field.

Moreover, recent studies employing advanced genomic technologies have highlighted the lungs’ diverse microbial environment, which is essential for optimal lung health [58] and serves as a defensive barrier against respiratory diseases. The dynamic interplay among the lung microbiome, pathogenic viruses, and host immunity is pivotal in dictating lung inflammation and immune responses [59]. There is increasing evidence to suggest that the interaction between the microbiota in the lungs and the host’s immune system is essential for maintaining a balanced immune system in the lungs.

Wang et al. reported that SPF mice were more vulnerable to acute inflammation and mortality post-influenza virus exposure compared to mice in natural environments [60]. Notably, the presence of commensal *S. aureus* significantly diminished influenza-mediated pulmonary immune damage, which is essential for resisting severe inflammatory responses. Zhang et al. leveraged LD_50_ and macro-genome sequencing analysis to pinpoint specific anti-influenza gut microbes and decipher the underlying mechanisms [50]. They observed alterations in the mice’s gut microbiota post-influenza infection and dynamically mapped gut microbiota shifts.

Furthermore, the increased prevalence of deterioration-related bacteria, such as *Haemophilus*, *Pseudomonas*, and *Moraxella*, has been associated with conditions like cystic fibrosis (CF), chronic obstructive pulmonary disease (COPD), and other persistent lung diseases [61, 62]. Mathieu et al. documented an elevated presence of Aspergillus in asthmatic patients’ lung microbiota [63]. They found that discrepancies in the lung microbiota components were evident between lung cancer patients and healthy individuals. Typically, alpha diversity was considerably higher in non-tumour lung tissues compared to tumorous counterparts.

Laroumagne et al. identified gram-negative bacteria, including *Haemophilus influenzae*, *Enterobacter* spp, and *Escherichia coli*, colonising lung cancer patients’ respiratory tracts [64]. This finding suggests that certain pathogens might disrupt the respiratory microbiota balance, thereby modulating disease onset and progression. Similarly, our study devised an H9N2 infection mouse model, revealing varying clinical manifestations in mice post-H9N2 influenza virus infection. Respiratory microorganisms from the mild disease group exhibited a protective response against influenza, enhancing mouse survival rates.

We found that histological and immunohistochemical analyses corroborated that mice treated with respiratory microorganisms from the mild disease group exhibited fewer lung pathological alterations and a reduced viral load. Through 16 s sequencing, we distinguished pronounced differences in the respiratory microbiota between severely and mildly infected mice post-influenza. The *Proteobacteria*, *Actinobacteria*, *Bacteroidota*, *Firmicutes*, and *Deinococcota* were dominant in groups M and S. *Firmicutes* showed reduced abundance in Group M relative to Group S. Groups M and S were dominated by *Pseudomonas* spp., *Ralstonia*, spp., *Stenotrophomonas* spp., *Empedobacter* spp., and *Aeromicrobium* spp.

*Pseudomonas* spp. were more abundant in group S than group M, whereas *Ralstonia* spp., *Stenotrophomonas* spp., *Empedobacter* spp., and *Aeromicrobium* spp. were more prevalent in group M. Marked differences between groups M and S were also observed for *Pseudomonas* spp, *Ralstonia* spp., and *Aeromicrobium* spp. Such variances in respiratory microorganisms between groups post-influenza infection might contribute to the diverse clinical manifestations following influenza infection.

Recent research on the lung microbiome has improved our understanding of the potential therapeutic targets found within commensal flora for an array of respiratory ailments [65]. Probiotics have gained widespread acceptance in treating infectious respiratory diseases due to their pronounced effects in bolstering the host’s immunity and countering pathogenic invasions [66]. Numerous studies confirm that introducing oral or nasal lactobacilli and various probiotics can recalibrate the respiratory tract’s innate immune response, thus bolstering resistance against influenza viruses. Additionally, several strains of *lactobacilli* (LAB) are known for invigorating the mucosal immune system and offering robust defence against *Streptococcus pneumoniae* infections [67–69]. Zhang et al. and Shida et al. established that probiotic blends, such as Clear Run yoghurt and *Lactobacillus casei*, fortify the respiratory tract. Their research particularly emphasised a reduction of infection occurrences, diminished duration, lessened severity, and, notably, enhanced immune markers [70, 71].

Furthermore, infant formula fortified with prebiotics and probiotics has effectively curtailed respiratory infections [72]. Tomita et al.’s animal study on RSV infection clarified that intranasal delivery of *Lactobacillus rhamnosus* heightened mice resistance to RSV exposure [73]. Our current research indicated that the mice treated with *Aeromicrobium camelliae* exhibited substantial resistance to severe influenza virus infections, which is evident through limited weight loss, diminished lung viral loads, and increased survival rates. Consequently, *Aeromicrobium camelliae* exhibits potential anti-influenza properties, marking it as a promising probiotic strain.

As we have shown, microbial byproducts are pivotal in shaping host health and specific disease manifestations [74]. For instance, gut-derived metabolites, such as succinate and itaconic acid, influence macrophage phagocytic activity and aid in the clearance of bacterial infection [75]. Steed et al. demonstrated that the inclusion of desamino-tyrosine (DAT, a flavonoid degradation derivative of intestinal bacteria) in drinking water shielded mice from the repercussions of influenza virus infections, particularly mortality and weight reductions [76]. In a separate investigation, Wypych et al. identified that cresyl sulfate (PCS), a culmination of L-tyrosine metabolism facilitated by the gut microbiota, safeguards mice from allergic respiratory inflammation [77]. Such revelations highlight the indispensable role of microbiota-synthesised metabolites in maintaining organismal health. Similarly, our analysis showed that mice administered with nasal drops of LysoPE showcased heightened resilience against the influenza virus.

Moreover, histopathological and immunohistochemical assessments revealed minimal pathological deviations and reduced lung viral concentrations, thus enhancing survival prospects. Utaipan T emphasised the efficacy of ursodeoxycholanoyl lysophosphatidyl ethanolamide in averting CD95 / FAS-induced fulminant hepatitis [78]. We suggest that certain compounds, specifically LysoPE, possess anti-inflammatory properties. However, further research is needed to understand the anti-inflammatory mechanism of LysoPE (16:0) fully.

In conclusion, we observed heterogeneity in the response to influenza infection in a mouse model. Moreover, we identified *Aeromicrobium* as a member of the respiratory microbiota that can protect against influenza infection. We demonstrate that the genus *Aeromicrobium* is elevated in abundance in the lungs following influenza virus infection, thereby protecting the host against the virus. Our study also represents a new way in which the respiratory microbiota can protect the host against infection, possibly through a novel interaction between the host and the respiratory microbiota. In addition to the gut microbiota, we also found that respiratory microbiota can be used as a novel biomarker to predict the severity and mortality of influenza patients, which may help provide a new idea for the precise treatment of influenza in the future.
